# Precise control of digital dental unit to reduce aerosol and splatter production: new challenges for future epidemics

**DOI:** 10.1186/s12903-024-03980-w

**Published:** 2024-02-10

**Authors:** Yuedi Yu, Xueling Wu, Yang Sun

**Affiliations:** 1 College of Dental Medicine, Columbia University, New York, NY 10032 USA; 2 Aaron Diamond AIDS Research Center, Columbia University Vagelos College of Physicians and Surgeons, New York, NY 10032 USA; 3 Department of Stomatology, Zhongshan Hospital, Fudan University, No. 180 Fenglin road, Shanghai, 200032 China

**Keywords:** Dental handpiece speed, Cooling condition, Droplet and aerosol splashing, SARS-CoV-2

## Abstract

**Background:**

During dental procedures, critical parameters, such as cooling condition, speed of the rotary dental turbine (handpiece), and distance and angle from pollution sources, were evaluated for transmission risk of the Severe Acute Respiratory Syndrome Coronavirus 2 (SARS-CoV-2), simulated by spiking in a plasmid encoding a modified viral spike protein, HexaPro (S6P), in droplets and aerosols.

**Methods:**

To simulate routine operation in dental clinics, dental procedures were conducted on a dental manikin within a digital dental unit, incorporating different dental handpiece speeds and cooling conditions. The tooth model was immersed in Coomassie brilliant blue dye and was pre-coated with 100 μL water spiked-in with S6P-encoding plasmid. Furthermore, the manikin was surrounded by filter papers and Petri dishes positioned at different distances and angles. Subsequently, the filter papers and Petri dishes were collected to evaluate the aerosol splash points and the viral load of S6P-encoding plasmid in aerosols and splatters generated during the dental procedure.

**Results:**

Aerosol splashing generated a localized pollution area extended up to 60 cm, with heightened contamination risks concentrated within a 30 cm radius. Significant differences in aerosol splash points and viral load by different turbine handpiece speeds under any cooling condition (*P* < 0.05) were detected. The highest level of aerosol splash points and viral load were observed when the handpiece speed was set at 40,000 rpm. Conversely, the lowest level of aerosol splash point and viral load were found at a handpiece speed of 10,000 rpm. Moreover, the aerosol splash points with higher viral load were more prominent in the positions of the operator and assistant compared to other positions. Additionally, the position of the operator exhibited the highest viral load among all positions.

**Conclusions:**

To minimize the spread of aerosol and virus in clinics, dentists are supposed to adopt the minimal viable speed of a dental handpiece with limited cooling water during dental procedures. In addition, comprehensive personal protective equipment is necessary for both dental providers and dental assistants.

## Background

In March 2020, Severe Acute Respiratory Syndrome Coronavirus 2 (SARS-CoV-2) outbreak emerged globally, marking the third outbreak following the 2003 Severe Acute Respiratory Syndrome and 2012 Middle East Respiratory Syndrome for human Coronavirus disease (COVID). SARS-CoV-2 caused varying degrees of fever and physical discomfort in young people. Moreover, elderly patients with underlying conditions experienced particularly severe symptoms and most succumbed to progressive respiratory failure. To curb the spread of the virus, governments worldwide implemented a series of social lockdown policies. The pandemic led to the collapse of medical systems in several regions and the closure of dentistry departments. Moreover, the global economies were hit hard.

SARS-CoV-2 is primarily transmitted through direct contacts by respiratory droplets and aerosols, with the upper air way and salivary gland serving as the early sites of infection [1, 2]. Larger droplets carrying SARS-CoV-2 deposit heavily in the upper respiratory tract, while smaller aerosols can directly enter the lungs, leading to Lower Respiratory Tract Infections. To date, this virus has evolved a variety of variants, and there is currently no specific treatment for these mutants. Therefore, it is of utmost importance to effectively prevent the spread of the virus from contaminated sources.

In dental practice, dental handpieces have the potential to spread viruses by aerosols that disperse into the surrounding environment. Despite the implementation of various protective measures by clinical staff, such as wearing N95 masks, facial barriers, gloves, and disposable protective clothing, the close proximity between patients and clinical staff makes it difficult to completely prevent viral transmission through droplets and aerosols. This poses a heightened risk of SARS-CoV-2 infection in the clinical setting during an ongoing pandemic. Consequently, it becomes crucial to urgently address the challenge to minimize the generation of droplets and aerosols that may carry SARS-CoV-2 during dental practice.

Although a great deal of research has been conducted on protective measures to reduce the risk of SARS-CoV-2 infection in dental clinics [3], there is limited evidence for source control strategies that aim to minimize aerosol production. In clinical practice, dental handpieces can generate varying droplets and aerosols under different dental handpiece rotational speeds and cooling conditions (air-water ratio). However, the dispersion pattern and viral load of these droplets and aerosols under different handpiece settings are still unknown. In this study, we used an advanced digital dental platform equipped with a precise controlling system to investigate the generation of droplets and aerosols during treatment process. By manipulating the rotational speed and cooling condition, we aimed to identify practical parameters that could effectively minimize the production of droplets and aerosols, thereby contributing to the control of SARS-CoV-2 transmission in clinical settings.

According to previous research studies, microbiological approaches are widely applied to mimic the transmission of viruses through droplets and aerosols. However, these studies have limitations. Bacterial particles do not fully represent the splash of viral particles as the transmission of these microbes differs from that of viruses in droplets and aerosols [4–6]. The viral Spike (S) protein is crucial for SARS-CoV-2 transmission and pathogenesis, making it a significant area of research. To address the wild-type S protein’s instability issue hindering large-scale production, scientists developed a mutated version called HexaPro (S6P) to improve stability, heat tolerance, and expression level [7, 8]. In this study, we used S6P as the model protein and spiked S6P-encoding plasmid in water to conduct a quantitative analysis of viral load at various clinical sites between patients and clinical staff. Furthermore, we evaluated the potential risk of infection based on the observed viral load. Based on the quantified results, we proposed practical strategies aimed at reducing the generation of droplets and aerosols during dental practice. These strategies could also contribute to reducing the risk of other viral infections, such as human immunodeficiency virus (HIV) and human papillomavirus (HPV) in dental setting.

## Materials and methods

The open clinic was located in the Center for Precision Dentistry of Columbia University College of Dental Medicine. Each procedure was performed under a digital dental unit (Planmeca Sovereign® Classic, Finland). The maxillary central incisors were prepared for 3 minutes using a dental handpiece to monitor the distribution of aerosols generated under different parameter settings in an open environment. To simulate the treatment scenario for COVID positive patients, Quantitative Real-Time Polymerase Chain Reaction (qPCR) was performed at the Aaron Diamond AIDS Research Center of Columbia University, to quantitatively investigate the generation of oral aerosols and the concentration of virus in aerosols and droplets.

### Aerosol particle scattering experiment

**Fig. 1:**
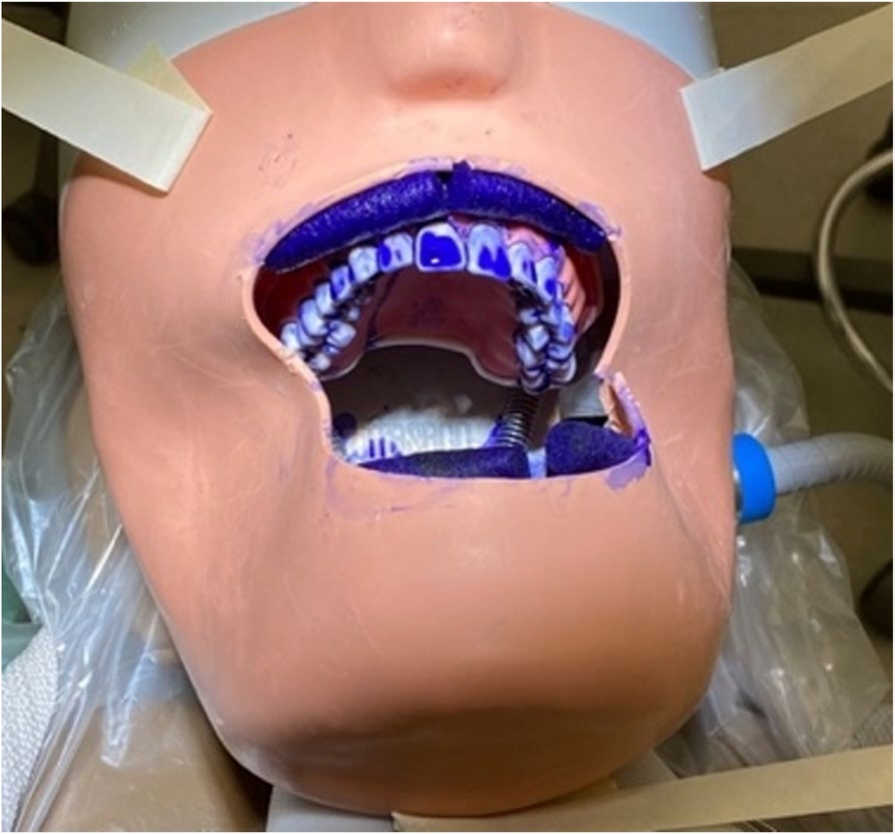
Demonstration of manikin oral cavity for the Coomassie blue dye experiment

Coomassie brilliant blue dye (R-250, Amresco, America) was used to simulate aerosols and droplets during dental treatment. The artificial teeth model was immersed in the dye for 1 hour. The position of the artificial teeth model was set at 73 cm from the ground to mimic the natural posture position [9]. As shown in Fig. 1, the left, right, upper, and lower cheeks of the dental manikin were filled and surrounded by four cotton rolls with a diameter of 10 mm to establish the dye repository. White cotton filter papers with the side length of 9.0 cm (Ahlstrom Chromatography Blotting Paper, Grade 222) were placed at different known positions at certain distances from the model to collect aerosols and droplets.

**Fig. 2:**
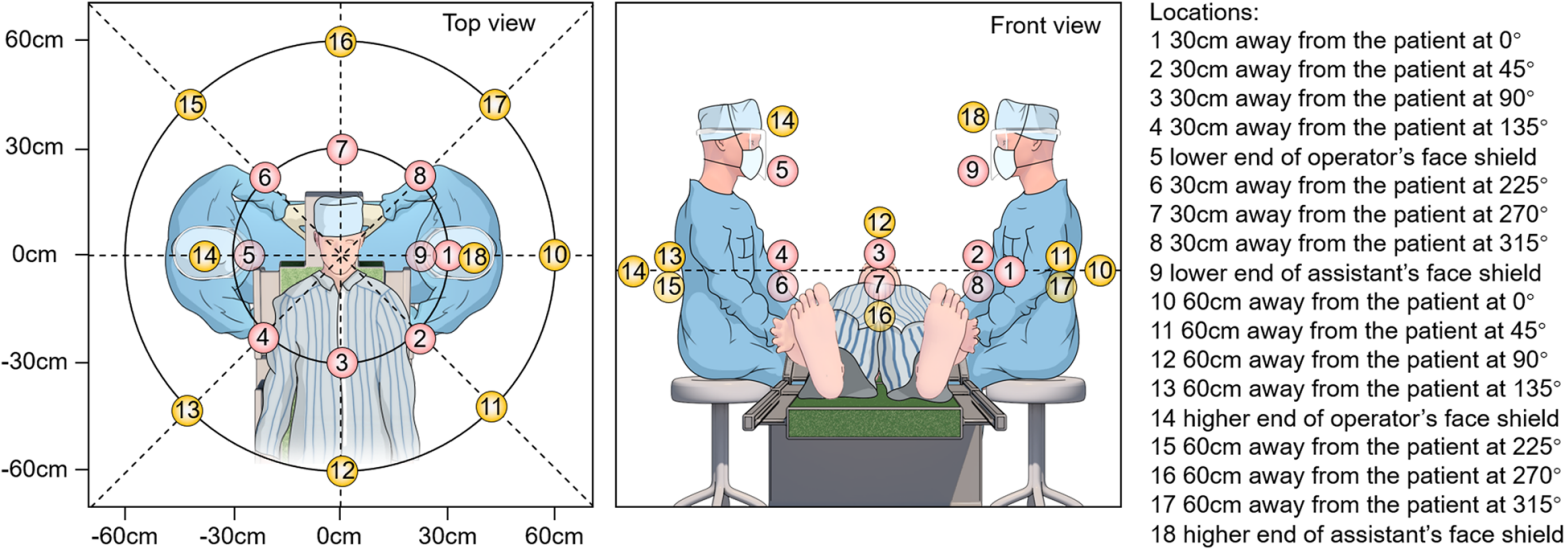
Schematic positions of operator, assistant, manikin during the mock dental procedure. The scheme exhibited top and front views with labeled collection positions and angles (angle was relative to facing the manikin)

**Fig. 3:**
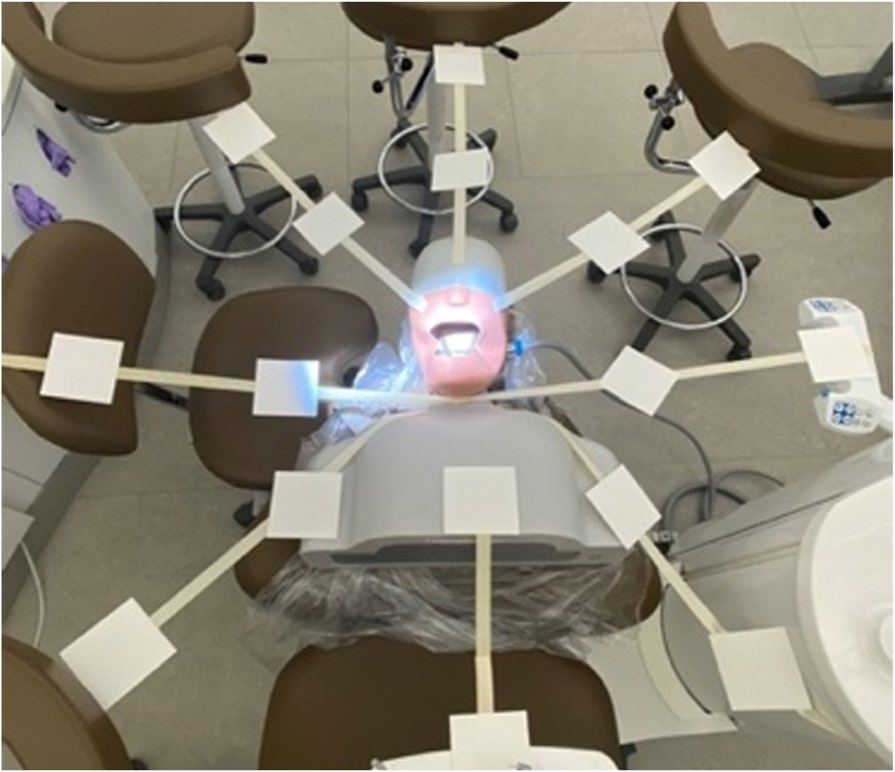
Distribution of filter papers in the plane position during the mock dental procedure (excluding the filter papers on the face shields of operator and assistant)

From the headrest, adhesive tapes were fixed in seven directions to support the cotton filter papers, laid out at 45° intervals, corresponding to 0°, 45°, 90°, 135°, 225°, 270°, 315° around the manikin. Filter papers were placed on these adhesive tapes with distances of 30 cm and 60 cm, in addition, two filter papers were respectively fixed on the face shield of both the operator and the assistant, with one paper fixed at the lower part of the face shield and another one fixed at the upper part of the face shield. In total, 18 filter papers were applied in the study (Figs. 2 and 3).

**Fig. 4:**
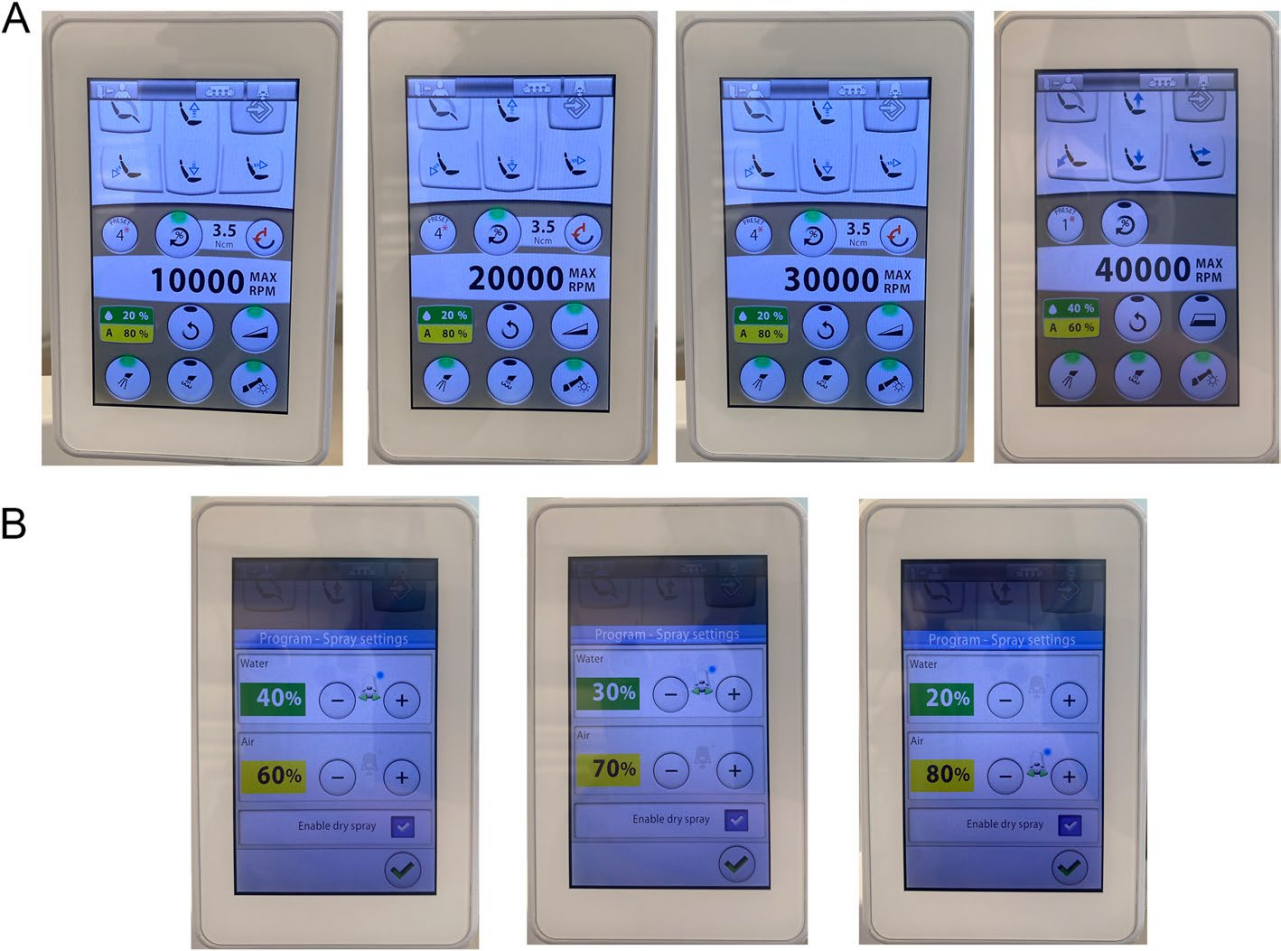
Digital dental unit with control panel where dental handpiece speed (**A**, top) and cooling condition (**B**, bottom) can be adjusted

An adjustable dental handpiece with precise and controllable speed and air-water ratio was used in this study. Specifically, four different handpiece speeds, 10,000, 20,000, 30,000, and 40,000 rpm were selected with three different air-water coolant conditions, 80% air - 20% water, 70% air - 30% water, and 60% air - 40% water (Fig. 4). In total, there were 12 different combinations of handpiece speed and air-water coolant setting (Table 1). After a 10-minute interval following the completion of the experiment, filter papers with precipitated droplets and aerosols were collected by tweezers to count the number of staining spots. Each experiment was repeated three times under the same experimental conditions. Before each experiment, the model was cleaned with 75% ethanol, the dental handpiece was disinfected with high temperature and pressure, and the platform was disinfected with ultraviolet light for 60 min.

**Table 1:** Pre-set digital dental unit operating parameters

Setting	Handpiece speed	Air-water coolant	Material	n
1	10,000 rpm	20% water, 80% air	Fluorescent dye: Coomassie Brilliant Blue R-250	3
2		30% water, 70% air		3
3		40% water, 60% air		3
4	20,000 rpm	20% water, 80% air		3
5		30% water, 70% air		3
6		40% water, 60% air		3
7	30,000 rpm	20% water, 80% air		3
8		30% water, 70% air		3
9		40% water, 60% air		3
10	40,000 rpm	20% water, 80% air		3
11		30% water, 70% air		3
12		40% water, 60% air		3

For the image analysis, all filter papers splashed with droplets and aerosols were counted by one operator twice and the average of counts was reported for accuracy. Overlapping spots were considered as multiple spots because they indicate distinct splatter events.

### Quantitative real-time PCR analysis

H_2_O with S6P plasmid (Addgene, rRID: Addgene_154754) was used to simulate the oral environment of COVID positive patients. 100 μL H_2_O with 1 ng/μL S6P plasmid was homogenously applied to the surface of the teeth model. The low-volume saliva ejector was attached to the head of the human body model and used throughout the experiment. Petri dishes with a diameter of 9 cm were used to collect aerosols and droplets at the positions of the filter papers. The PCR primers for S6P plasmid were: Forward, 5’GATTTGCCTCCGTTTACGCC3’, Reverse: 5’AGGTAGTTGTAGTTGCCGCC3’. The volumes of collected aerosol samples were quantified with a pipette, and the S6P plasmid in each sample was quantitatively analyzed using a qPCR kit (4,444,556, Thermo Fisher Scientific, America), with 5′(6FAM)ATTGTGTGGCCGACTACTCCGTGCTGT(BHQ1)3′. The qPCR was initiated at 95 °C for 2 minutes, followed by 50 cycles of 95 °C for 3 seconds and an annealing-extension step at 62 °C for 30 seconds.

### Statistical analysis

All quantitative data are expressed as mean ± standard deviation (SD). T-test is used for comparison between two groups, and one-way analysis of variance (ANOVA) is used for the analysis of more than two groups using GraphPad Prism 7.0 Software (USA). *P* value less than 0.05 is considered statistically significant (*P < 0.05, **P < 0.01, ***P < 0.001), and *P* > 0.05 means no statistical significance (ns).

## Results

### Coomassie brilliant blue dye analysis

**Fig. 5:**
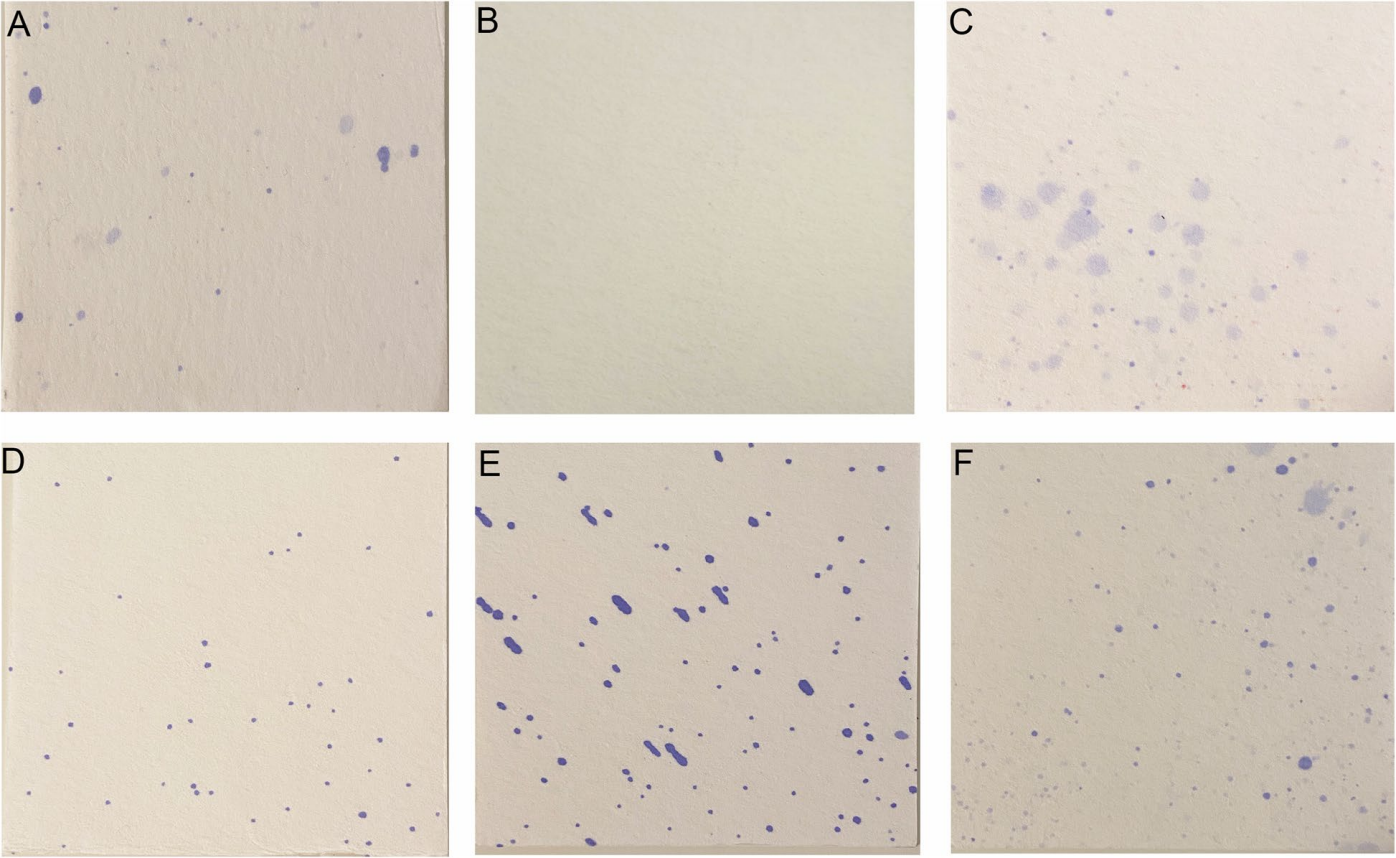
Counting the colored points on square filter papers: **A** at 30 cm 0° under 10,000 rpm and 80% air: 20% water; **B** at 60 cm 0° under 30,000 rpm and 60% air: 40% water; **C** at 30 cm 180° under 20,000 rpm and 60% air: 40% water; **D** at 30 cm 0° under 40,000 rpm and 80% air: 20% water; **E** at 30 cm 45° under 40,000 rpm and 70% air: 30% water; **F** at 30 cm 180° under 30,000 rpm and 80% air: 20% water

Filter papers were collected for image analysis, and all droplets and aerosols were counted for the analysis. As shown in Tables 2 and 3, the distribution of droplets and aerosols was examined under four different conditions, including distance, angle, handpiece rotational speed, and air-water ratio. Figure 5 displayed the numbers and distributions of droplets and aerosols from specific positions and angles.

**Table 2:** Average amount of aerosol and splatter at different positions with different settings at 30 cm

Distance	Position	Contaminated Spots											
		10,000 rpm			20,000 rpm			30,000 rpm			40,000 rpm		
		20% water	30% water	40% water	20% water	30% water	40% water	20% water	30% water	40% water	20% water	30% water	40% water
30 cm	0°	8.9	–	–	14	10.2	22.9	15.3	8.9	136.2	19	14	152
	45°	–	–	–	–	6.4	0.0	2.5	–	–	1.0	105.0	58.0
	90°	–	–	–	–	1.3	3.8	–	–	5.1	14.0	11.0	54.0
	135°	11.5	–	–	–	–	2.5	–	–	2.5	3.0	1.0	2.0
	180° = O-FS	25.5	39.5	91.7	38.2	64.9	87.9	58.6	71.3	168.1	43.0	214.0	118.0
	225°	15.3	15.3	15.3	7.6	3.8	2.5	36.9	5.1	8.9	32.0	38.0	78.0
	270°	1.3	–	–	2.5	10.2	1.3	8.9	11.5	10.2	3.0	14.0	1.0
	315°	–	3.8	8.9	2.5	24.2	2.5	29.3	21.6	39.5	29.0	9.0	12.0
	A-FS	–	1.3	–	–	–	–	–	1.3	1.3	7.0	42.0	4.0
	Mean	6.9	7.5	20.4	7.4	13.4	13.7	16.8	13.6	41.3	16.8	49.8	53.2
	St. Dev	9.1	13.0	33.6	12.5	20.7	28.7	20.7	22.7	64.4	15.0	69.2	54.8

*O-FS* Operator’s Facial Shield, *A-FS* Assistant’s Facial Shield

**Table 3:** Average amount of aerosol and splatter at different positions with different settings at 60 cm

Distance	Position	Contaminated Spots											
		10,000 rpm			20,000 rpm			30,000 rpm			40,000 rpm		
		20% water	30% water	40% water	20% water	30% water	40% water	20% water	30% water	40% water	20% water	30% water	40% water
60 cm	0°	–	–	–	–	–	–	–	–	–	–	–	–
	45°	–	–	–	–	–	–	–	–	–	–	1.0	–
	90°	–	–	–	–	–	–	–	–	–	–	–	1.0
	135°	–	–	–	–	–	–	–	–	1.3	–	–	–
	180° = O-FS	–	1.3	1.3	–	–	–	–	–	–	–	–	–
	225°	–	–	–	–	–	2.5	–	–	–	–	–	2.0
	270°	–	–	–	–	–	–	–	–	–	–	–	–
	315°	–	–	–	–	–	–	–	–	–	–	–	–
	A-FS	–	–	–	–	–	1.3	–	–	–	–	–	–
	Mean	–	0.1	0.1	–	–	0.4	–	–	0.1	–	0.1	0.3
	St. Dev	–	0.4	0.4	–	–	0.9	–	–	0.4	–	0.3	0.7

*O-FS* Operator’s Facial Shield, *A-FS* Assistant’s Facial Shield

#### The droplets and aerosols were significantly different at 30 cm and 60 cm from the pollution source

Most droplets and aerosols were observed at 30 cm from the pollution source, with the numbers and distributions decreasing as the distance increased. At 60 cm, the droplets and aerosols appeared at any angle randomly. Moreover, at 30 cm, the numbers and distributions of droplets and aerosols increased with higher handpiece rotational speeds. Additionally, significant differences in the numbers and distributions of droplets and aerosols were observed at 30 cm and 60 cm from the source under 12 different combinations of dental handpiece speed and air-water coolant parameters, as shown in Table 4, *P < 0.05.* According to the results, limited droplets and aerosols can be observed at 60 cm from the source; however, operators and assistants should still maintain effective protection gears within this range.

**Table 4:** Comparing average amount of aerosol and splatter produced at 30 cm and 60 cm around the manikin

Setting	30 cm	60 cm	St. Error Difference	*p*-Value
10,000 rpm & 20% water	6.9	–	3.036	0.018
10,000 rpm & 30% water	7.5	0.1	4.588	0.022
10,000 rpm & 40% water	20.4	0.1	10.370	0.049
20,000 rpm & 20% water	7.4	–	4.153	0.048
20,000 rpm & 30% water	13.4	–	8.394	0.049
20,000 rpm & 40% water	13.7	0.4	11.140	0.048
30,000 rpm & 20% water	16.8	–	6.895	0.013
30,000 rpm & 30% water	13.6	–	7.580	0.039
30,000 rpm & 40% water	41.3	0.1	21.480	0.037
40,000 rpm & 20% water	16.8	–	5.008	0.002
40,000 rpm & 30% water	49.8	0.1	22.760	0.010
40,000 rpm & 40% water	53.2	0.3	18.280	0.005

#### The droplets and aerosols were mainly deposited at the positions of operator and assistant rather than other positions

**Fig. 6:**
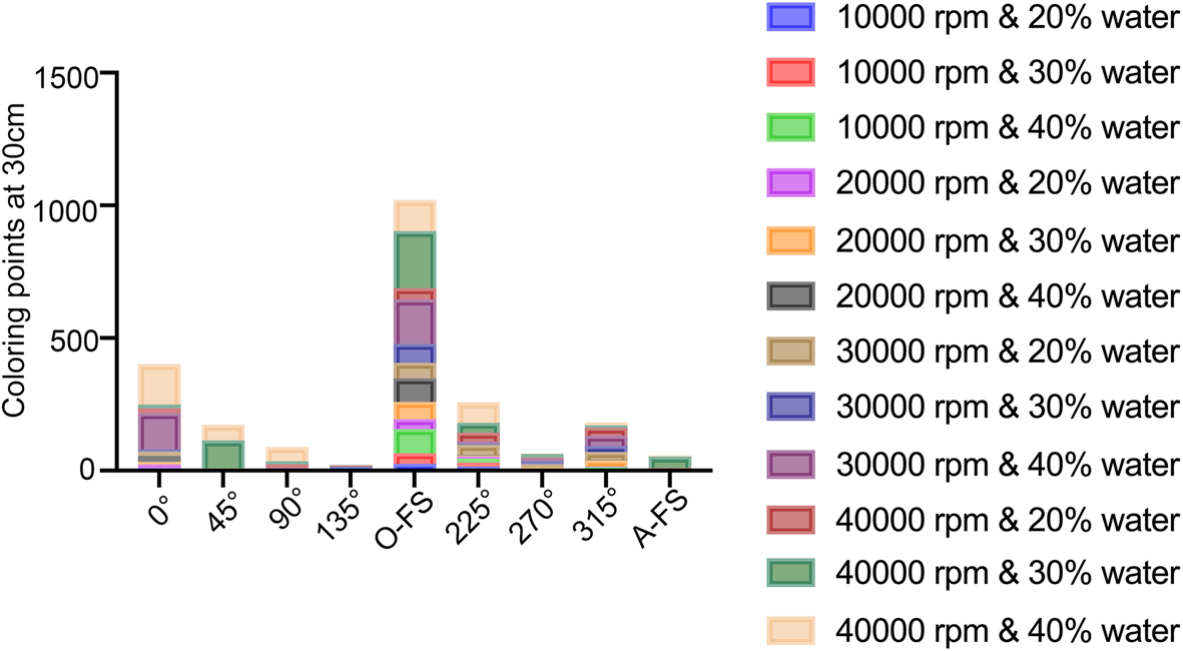
Amount of splatter points at 30 cm from the contamination source under different cooling conditions and dental handpiece speeds (O-FS, lower end of operator’s face shield; A-FS, lower end of assistant’s face shield)

Since limited droplets and aerosols appeared at 60 cm from the source, the droplets and aerosols at 30 cm from the source were used for the following statistical analysis. Under 12 different combinations of dental handpiece speed and air-water coolant parameters, the angles of droplets and aerosols were statistically analyzed. As shown in Fig. 6, 1020.7 droplets and aerosols appeared at the position of operator from 180^o^, and 401.4 droplets and aerosols were observed at the position of assistant’s left arm from 0°. Additionally, 258.7 droplets and aerosols appeared on the left arm of operator from 225°, and 182.3 droplets and aerosols were observed on the right arm of assistant from 315° (Fig. 6).

**Fig. 7:**
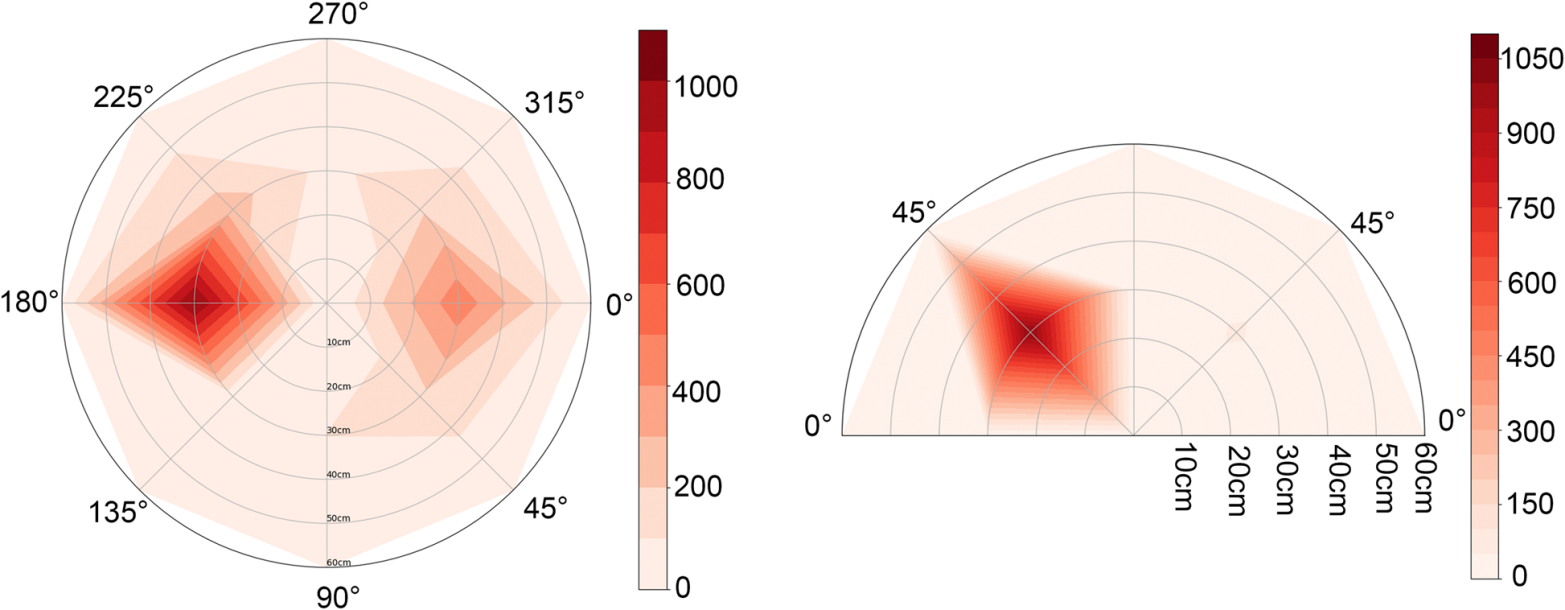
Heatmaps of distribution of aerosol and splatter around the patient. For each coordinate, the summation of average value recorded under each operating parameters setting was used as this was deemed most clinically relevant

To visualize the distribution of droplets and aerosols, a splash heat map was generated by Python software. From the top view, majority of droplets and aerosols were concentrated on both the inclined surface and the plane surface, corresponding to the positions of the operator and the assistant, respectively, at a distance of 30 cm from the pollution source. The angles associated with these positions were 180° and 0°, respectively. From the front view, the face shield of both the operator and the assistant were found to be the primary sites where aerosols and droplets were concentrated. As a result, the operator displayed a higher level of contamination risk compared to the assistant (Fig. 7).

#### The droplets and aerosols increased with raising dental handpiece speeds under fixed air-water ratios

When the air-water ratio respectively was fixed at 80%: 20, 70%: 30%, or 60%: 40%, the precipitation points of aerosols and droplets were significantly different at different dental handpiece speeds (*p < 0.05*) (Table 5). When the air-water ratio was fixed, the precipitation points of aerosols and droplets increased with the raising turbine handpiece speeds.

**Table 5:** At fixed air-water ratio, average amount of aerosol produced under different handpiece speed was compared

Distance	Air-water coolant	Handpiece speed	Av. Aerosol	*p*-Value
30 cm	20% water	10,000 rpm 20,000 rpm 30,000 rpm 40,000 rpm	6.9 7.4 16.8 16.8	0.031
	30% water	10,000 rpm 20,000 rpm 30,000 rpm 40,000 rpm	7.5 13.4 13.6 49.8	0.033
	40% water	10,000 rpm 20,000 rpm 30,000 rpm 40,000 rpm	20.4 13.7 41.3 53.2	0.020

#### Air-water ratio did not influence droplets and aerosols under fixed dental handpiece speeds

When the dental handpiece speed was fixed, varying air-water ratios did not influence the overall precipitation points of droplets and aerosols (*p* > 0.05) (Table 6). Considering that most precipitation points occurred at 0° and 180° angles, further analysis was conducted specifically on the precipitation points at these angles while keeping the turbine handpiece speed fixed. As shown in Table 7, the precipitation points were significantly different when the handpiece speed was 10,000 rpm (*p* = 0.036), 30,000 rpm (*p* = 0.035), and 40,000 rpm (*p* = 0.040) at 180° position, corresponding to the position of the operator. Additionally, the precipitation points were statistically different when the handpiece speed was 10,000 rpm (*p* = 0.024) at 0° position, corresponding to the position of the assistant.

**Table 6:** At fixed speed, average amount of aerosol produced under different air-water ratios was compared at 30 cm

Distance	Handpiece speed	Air-water coolant	Av. Aerosol	*p*-Value
30 cm	10,000 rpm	20% water 30% water 40% water	6.9 7.5 20.4	0.223
	20,000 rpm	20% water 30% water 40% water	7.4 13.4 13.7	0.234
	30,000 rpm	20% water 30% water 40% water	16.8 13.6 41.3	0.244
	40,000 rpm	20% water 30% water 40% water	16.8 49.8 53.2	0.079

**Table 7:** At fixed speed, average amount of aerosol produced under different air-water ratios at 0 ° and 180 ° was compared

Distance	Position	Handpiece speed	Air-water coolant	Av. Aerosol	*p*-Value
30 cm	180°	10,000 rpm	20% water 30% water 40% water	25.5 39.5 91.7	0.036
		20,000 rpm	20% water 30% water 40% water	38.2 64.9 87.9	0.425
		30,000 rpm	20% water 30% water 40% water	58.6 71.3 169.1	0.035
		40,000 rpm	20% water 30% water 40% water	43.0 214.0 118.0	0.040
	0°	10,000 rpm	20% water 30% water 40% water	8.9 - -	0.024
		20,000 rpm	20% water 30% water 40% water	14 10.2 22.9	0.173
		30,000 rpm	20% water 30% water 40% water	15.3 8.9 136.2	0.243
		40,000 rpm	20% water 30% water 40% water	19 14 152	0.191i

### Quantitative real-time PCR analysis

**Fig. 8:**
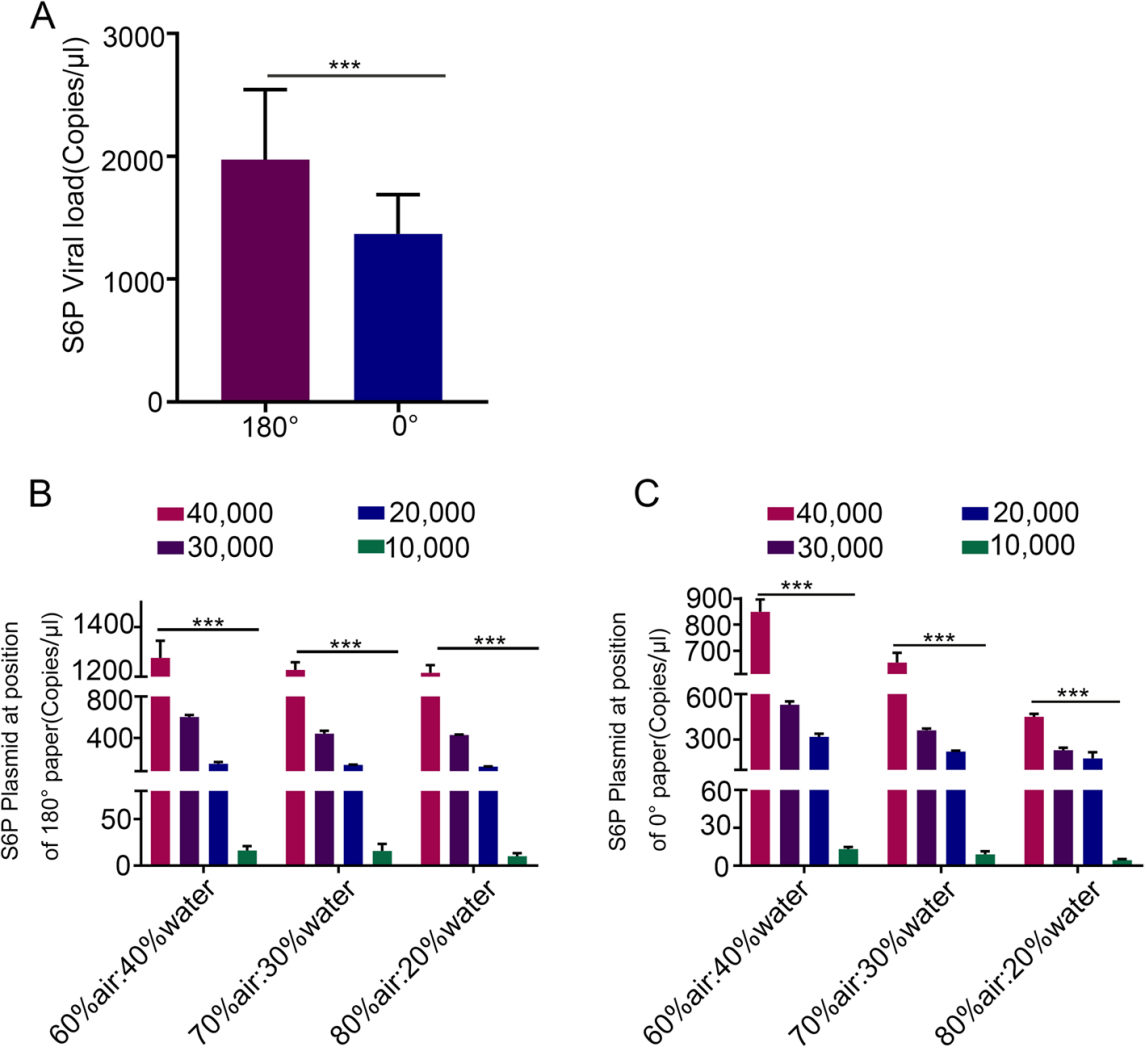
Viral load quantified by fluorescence quantitative PCR under different conditions

Given the high concentration of droplets and aerosols observed at the 0° and 180° positions at 30 cm from the source, these specific areas were selected for the quantification of virus load simulated with the S6P plasmid in collected samples. 12 different combinations of dental handpiece speed and air-water coolant parameters were utilized, with each combination being processed for a duration of 1 minute. The total droplets and aerosols were collected and averaged based on the results of twelve experiments. As shown in Fig. 8A, the S6P plasmid viral load of droplets and aerosols was significantly different at 0 ° and 180 ° (*p < 0.001)*. Furthermore, the S6P plasmid viral load exhibited statistically significant differences among the various dental handpiece speeds when the air-water coolant was kept constant. The highest viral load was observed when the air-water ratio was 60%: 40% with a dental handpiece speed of 40,000 rpm. Under these conditions, the average viral load was 1.28 × 10^3^ copies/μL at the operator’s position, and the average viral load was 8.49 × 10^2^ copies/μL at the assistant’s position. On the other hand, the lowest viral load was observed when the dental handpiece speed was 10,000 rpm, regardless of the air-water ratios. With a dental handpiece speed at 10,000 rpm, the average viral load was 14.16 copies/μL at the operator’s position and 8.93 copies/μL at the assistant position. Compared to the assistant’s position (0 °), a higher S6P viral load can be found in the operator’s position (180 °), as shown in Fig. 8B and C.

## Discussion

There are three main routes of transmission for infectious aerosols in dentistry, which include direct contact, surface contact, and instrument splashing [10]. The U.S. Centers for Disease Control and Prevention (CDC) defines aerosol as a suspension containing tiny (< 5 μm) inhalable particles or droplets in the air [11]. The World Health Organization (WHO) indicates that dental equipment could produce aerosols and droplets, increasing the transmission risk of infectious diseases [12]. According to reports, most of the visual aerosol particles are generated by rotary dental handpieces, followed by ultrasonic dental scalers [13]. Aerosols have the capacity to disperse through the air and readily adhere to various objects within the clinic, including tables, floors, computers, and medical staff. Unfortunately, although the large-capacity vacuum pump can reduce the propagation by more than 75%, particles smaller than 2.0 μm cannot be removed. In the context of aerosol mitigation strategies, Zhu et al.’s findings suggest that external aerosol scavenger units not only have a limited ability to eliminate aerosols but, more concerning, they may also redirect aerosol movement, thereby potentially heightening the risk of infection within the dental clinic [14]. Supplemental internal evacuation systems prove effective only in specific situations, depending on the tooth being operated on. Therefore, it is challenging to completely eradicate the transmission of aerosols [15]. According to reports, the most heavily contaminated areas with aerosols are the face and chests of medical staff, with no contamination observed beyond a three-meter distance from the source. Therefore, it would be meaningful to effectively manage and control the source of aerosol transmission.

Currently, the operating principle of clinically used dental turbine handpieces is driven by compressed air to rotate while avoiding overheating during the cutting process, equipped with an air-water spray cooling device [16]. During the rotation driven by compressed air, the cooling water is splashed around. Previous studies demonstrated that the contamination level generated by high-speed handpieces is significantly higher than that by low-speed handpieces [17, 18]. However, those studies only considered the single factor of handpiece speed and overlooked other factors that could influence the generation of aerosols. Annika Johnson et al. confirmed that traditional assistant-based irrigation and self-irrigating drills have no difference on the splashes produced, and the study also proved that hydrogen peroxide solution can produce a larger splash area than saline. However, the study did not consider the effect of different air-water ratio in the self-irrigating drills on the splash propagation [19]. For instance, increasing the speed of rotary handpiece usually requires a higher volume of water and air, resulting in an increased potential source of contamination. Hence, compressed air and cooling water of the dental handpiece may play a role in generating aerosols. Furthermore, Han, Pingping, et al. utilized high-speed and low-speed air turbine handpieces from different brands, which might introduce inherent variations in design and performance, thus affect study outcomes [18]. Also, previous studies have not yet discussed the optimal parameters for rotary speed and air-water ratios that can effectively minimize aerosol transmission [17, 18]. In comparison, we utilized a digital dental chair (Planmeca Sovereign® Classic, Finland) at Columbia University College of Dental Medicine Center for Precision Dentistry. This advanced equipment allows for precise adjustments of the handpiece speed and air-water ratio. Throughout the study, we consistently used the same dental handpiece and modified its settings via the control panel. This consistent methodology enabled accurate assessment of the impact of the handpiece on aerosol and splatter generation within the dental operatory and provided practical strategies for reducing droplets and aerosols during dental treatment.

Previous studies have identified several methods for collecting aerosols and splashes, such as instrumental, optical [12], filter paper [20], and spectroscopic methods [13, 21]. These techniques can analyze the collected splatter and aerosols using microbiological methods to determine their shape, size, and fluorescence intensity. However, instrumental methods can measure particle concentration but are limited to particles of a fixed size, making it challenging to discern viral particles [22, 23]. Similarly, filter paper, optical, and spectroscopic methods are unable to measure particle concentration, providing only droplet counts [9, 20, 24, 25]. Microbiological methods typically prioritize the detection of alpha-hemolytic streptococci or anaerobes, often neglecting viruses [6, 10]. The latest research utilized state-of-the-art experimental fluid mechanics tools to detect the number and the transmission speed of aerosol droplets through the advanced high-speed imaging techniques and optical flow tracking velocimetry. The initial velocity of these droplets can be quite significant, typically ranging between 1 m/s and 2.6 m/s [14, 26, 27].

Those studies concentrated on analyzing the properties of aerosol generation at a constant rotational speed, noting that the transmission path of aerosols varied with the turbine’s direction. In contrast, the purpose of our research is to find the best combination of generating less aerosol by precisely adjusting the rotation speed of the dental handpiece and the air-water ratio, so as to explore the methods of protecting oral hygiene professionals [14, 26, 27]. In this study, a simulated clinical working environment was established, and the transmission of aerosols and droplets was observed from various directions and angles on patients and medical staff. A significant concentration of aerosols and droplets was observed within 60 cm of the contamination source, with the highest concentration observed within 30 cm. Limited aerosols and droplets were found at 30–60 cm from the source. A previous study also demonstrated the greatest level of contamination from a dental procedure within a 1-ft radius of the source, diminishing at 2 ft [28]. Furthermore, our findings indicate that the operator and assistant positions displayed a higher degree of contamination when compared to other positions. Additionally, there was a notably greater level of contamination observed around the operator in comparison to the assistant. In the operator area, the maximum contamination was on the operator’s face shield, followed by the left arm, and the right arm of the operator exhibited minimal contamination. In the assistant area, the maximum contamination was on the left arm of assistant, followed by the right arm. The face shield of the assistant exhibited minimal contamination. Veena et al. reported that the right arm of the operator displayed the most contamination [28], while the assistant area was consistent with our results. However, a recent study has presented contrasting findings, revealing that the highest concentration of deposited splatters is primarily located on the patient’s chest, followed by the assistant’s face shield [19]. While there is no unanimous agreement regarding the exact location with the highest contamination within the operatory, it is evident that operators, assistants, and patients all face potential exposure to splatters and aerosol contaminants. Additionally, Zhu et al. revealed that the use of barriers can significantly reduce aerosol and splatter levels [14]. These findings altogether underscore the importance and effectiveness of implementing personal protective equipment (PPE) in dental settings.

When the air-water ratio was fixed, increase in dental handpiece speed significantly raised the precipitation amounts of droplets and aerosols. However, the air-water ratio variations did not have a significant effect on the overall amount of collected splatters and aerosols. One plausible explanation might be that an increase in water proportion enhances the weight of the splash droplets, leading to a reduced travel distance. As splatters were only captured at a distance of 30 cm or more, the heavier, shorter-traveling droplets might fall closer to the source. This missed capture of droplets could explain why changes in the water proportion do not appear to impact the measured quantities of splatter and aerosols. The detection limit, therefore, may not represent the full scope of splatter distributions, particularly for droplets that fall within a shorter radius due to increased weight from higher water content. When dental handpiece speeds were fixed at 10,000, 30,000, and 40,000 rpm, the precipitation amounts of droplets and aerosols increased with decreasing air-water ratios at the position of the operator. Therefore, besides the proper protection, it is necessary to use a large suction aspirator at the position of the operator. However, at the assistant side, the precipitation amounts of droplets and aerosols increased with decreasing air-water ratios only when the turbine handpiece speed was at 10,000 rpm. These results demonstrate that reducing the turbine handpiece speed and increasing the air-water ratios can effectively reduce the precipitation amounts of droplets and aerosols in clinical setting.

In addition to the commonly used capturing fluorescein dye with filter papers, we innovatively replaced the filter paper with petri dish to collect splattered liquid drops containing S6P-encoding plasmid to quantify viral load. However, in previous aerosol research methods, S6P plasmids were not introduced into the experiments [14, 19, 26, 27]. We then analyzed the copy number of the S6P plasmid through quantitative PCR, enabling the assessment of viral concentration and transmission. In prior research, the assessment of aerosol contamination has primarily centered on bacteria [6, 10]. However, bacteria are relatively large so they can only provide an indication of the extent of droplet splatter rather than the finer aerosol particles. Virus can also potentially have a wider transmission range compared to bacteria due to its smaller size [10]. Zemouri et al. revealed that the bacteria-containing aerosols generated by dental treatments were mainly distributed around patient’s head, which align with our results on aerosol distribution [25].

During the COVID-19 outbreak, aerosols containing SARS-CoV-2 virus particles were a major source of contamination in dental clinics. The size of SARS-CoV-2 is 0.005–0.2 μm [29]. The spread of virus particles is different from that of larger bacterial particles, as they can be easily transmitted via aerosols and remain suspended in the air for several hours. SARS-CoV-2 can survive for up to 72 hours on the surface of stainless steel and plastic, up to 24 hours on the surface of cardboard, and up to 4 hours on the surface of copper. Moreover, it can survive up to 1 day on clothes, up to 7 days on the outer layer of surgical masks, and up to 2 weeks at low temperatures [30, 31]. Similar to the SARS virus, SARS-CoV-2 recognizes human ACE2 protein by viral spike protein, internalizing into cells.

According to previous study, medical staff could be exposed to 0.014–0.12 μl of saliva after 15 minutes of treatment with a high-speed dental handpiece [32]. The saliva of infected patients contains the SARS-CoV-2 virus, with a median viral load of 3.3 × 10^6^ copies/mL [33]. In this study, the viral load was significantly different at various distances and directions.

Recent computer simulations by Jonathan Komperda et al. provides valuable insight into the field [34]. Nonetheless, our study was carried out in a single operatory, providing a more direct and realistic reflection of actual clinical conditions as opposed to those derived from simulations set within the complex environment of a large dental clinic. Moreover, computer or numerical simulation software necessitates specific parameters for calculations. The omission of crucial parameters, including the rotational speed of dental turbine handpieces, air-water ratio, and aerosol splashing distance, may significantly impact the study outcomes. The primary objective of our study was to closely replicate actual clinical procedures, thereby enabling the experimental results and findings applicable to dental practice and instrumental in determining necessary parameters to for future modeling efforts.

In summary, this study thoroughly examined the impact of dental chair operating parameters, including rotational speed, air-water ratio, and distance and angle from the pollution source, on the distribution of SARS-CoV-2 aerosol, providing practical insights for dental treatment. Our findings indicated the transmission capacity of aerosols containing SARS-CoV-2 virus decreased with reducing rotational speed, under any fixed air-water ratios tested. However, this study still has some limitations. Firstly, the study concentrated exclusively on maxillary central incisors, but variations in tooth position and consequent adjustments in operator position may influence splatter patterns and outcomes. Secondly, the influence of treating duration on viral load remains unknown. Although we simulated the dental treatments by aerosol splash experiment, the effectiveness and accuracy of the aerosol splash experiment still need to be further confirmed in real clinical settings.

## Conclusions

In this study, the spatial spreading pattern, including the distance and angles, and virus load of S6P plasmid were detailly evaluated under different rotational speeds and air-water ratios of the dental handpiece. Our qualitative and quantitative analyses revealed that the positions of the dentist and assistant exhibited higher virus loads compared to other positions, with the dentist’s position showing the highest virus load. Furthermore, we observed a reduction in virus load of aerosols with decreasing dental handpiece rotational speed. This study provides a valuable theoretical foundation for effectively preventing the transmission of various viruses, including HIV, HPV, Influenza, the new COVID-19 Omicron variant (XBB.1.16), and other potential future viruses.

## Data Availability

The datasets used and/or analyzed during the current study are available from the corresponding author on reasonable request.
